# Asymptomatic infection with American cutaneous leishmaniasis: epidemiological and immunological studies

**DOI:** 10.1590/0074-02760160138

**Published:** 2016-10

**Authors:** Fernando J Andrade-Narvaez, Elsy Nalleli Loría-Cervera, Erika I Sosa-Bibiano, Nicole R Van Wynsberghe

**Affiliations:** Universidad Autónoma de Yucatán, Centro de Investigaciones Regionales Dr Hideyo Noguchi, Laboratorio de Inmunología, Mérida, Yucatán, México

**Keywords:** American cutaneous leishmaniasis, asymptomatic infection

## Abstract

American cutaneous leishmaniasis (ACL) is a major public health problem caused by vector-borne protozoan intracellular parasites from the genus Leishmania, subgenera Viannia and Leishmania. Asymptomatic infection is the most common outcome after Leishmania inoculation. There is incomplete knowledge of the biological processes explaining the absence of signs or symptoms in most cases while other cases present a variety of clinical findings. Most studies of asymptomatic infection have been conducted in areas of endemic visceral leishmaniasis. In contrast, asymptomatic ACL infection has been neglected. This review is focused on the following: (1) epidemiological studies supporting the existence of asymptomatic ACL infection and (2) immunological studies conducted to understand the mechanisms responsible for controlling the parasite and avoiding tissue damage.

The term Leishmaniasis refers to a group of vector-borne diseases caused by obligate intracellular protozoan parasites of the genus *Leishmania*, belonging to the family Trypanosomatida, order Kinetoplastida. *Leishmania* is transmitted to humans by sand flies of the genus *Phlebotomus* in the Old World and *Lutzomyia* in the New World. *Leishmania* infections have been reported in 98 countries on all continents. The disease is endemic in tropical and subtropical regions. There are an estimated 1.3 million new cases worldwide annually, of which 1 million are cutaneous leishmaniasis (WHO 2010, 2015, Alvar et al. 2012).

American cutaneous leishmaniasis (ACL) is caused by numerous species of the *Leishmania* or *Viannia* subgenera. The most prevalent species are *L.* (*V.*) *braziliensis* followed by *L.* (*L.*) *amazonensis*, *L.* (*V.*) *guyanensis* and *L.* (*V.*) *panamensis*. However, other species such as *L.* (*L.*) *mexicana*, *L.* (*L.*) *pifanoi*, *L.* (*L.*) *venezuelensis*, *L.* (*V.*) *peruviana*, *L.* (*L.*) *shawi* and *L.* (*V.*) *lainsoni* are present mostly in the Amazon Region and Central America (Goto & Lindoso 2010).

After parasite inoculation, the infection outcomes range from asymptomatic (absence or unapparent signs and symptoms) to clinically overt disease. The former is by far the most common outcome in endemic areas of ACL. For *Leishmania* spp. and other infectious agents, we have incomplete knowledge of why in most cases there are no signs and symptoms while other cases present a wide variety of clinical findings. Multiple factors involved in clinical infection have been studied at length including the parasite species and its virulence factors, as well as the host immune status and genetic determinants. Nonetheless the potential role of those factors has been neglected in the setting of asymptomatic ACL infection. Indeed, no review of ACL asymptomatic infection has been published in the literature to date.

Most studies on asymptomatic infection were performed in endemic areas of visceral leishmaniasis (VL) in both the New and Old World. It has been argued that the identification of potential asymptomatic carriers in VL endemic areas could be useful to (i) evaluate their role as potential reservoirs of the parasite; (ii) determine the effectiveness of control measures; (iii) evaluate the risk of transmission through blood donation or vertical transmission; and (iv) survey the population at risk of developing the disease (immunosuppressed subjects) that could be identified early (Bañuls et al. 2011, Michel et al. 2011, Sahgrouni et al. 2012).

This is the first review of asymptomatic ACL infection. Based on the literature available, it is focused on epidemiological knowledge supporting its existence and on immunological studies conducted to understand the mechanisms responsible for controlling the parasite and avoiding tissue damage.

*Asymptomatic infection* - For many infectious diseases, the early disease process involves pathological changes that occur unbeknownst to the individual. This stage of subclinical disease extends from the time of exposure to the onset of disease symptoms and is usually called the incubation period for acute infectious diseases and latency for chronic diseases. Nonetheless, persons with unapparent or undiagnosed infections may be able to transmit infection to others. People who are infectious but have no signs and symptoms are called asymptomatic carriers (CDC 2006).

There are no consensus criteria for diagnosing asymptomatic ACL infection due to the lack of methods to differentiate immunologically sensitising exposure from persistent *Leishmania* infection. The Montenegro skin test (MST) positive response is by far the most useful marker for asymptomatic ACL infection. Thus, the term asymptomatic ACL infection is used to refer to those individuals living in endemic areas of ACL without signs and symptoms and with a positive MST.

*Montenegro skin test* - Montenegro (1926) developed a skin test through the intradermal application of phenolized cultured promastigotes of *Leishmania* as antigens. It was developed to facilitate the diagnosis of ACL in endemic areas of South America where parasite visualisation was difficult. Since then, the MST has been widely used to support the diagnosis of CL in the New World. The MST, also known as the leishmanin test (similar to the tuberculin test), is categorised as a delayed type IV hypersensitivity (DTH) reaction (Turk 1979). MST reactivity induced by asymptomatic or symptomatic infection is typically long-lasting and does not discriminate between present or past infection. Nevertheless, it has been very useful for epidemiological studies to determine endemicity measured by the allergic index, *i.e.*, the percentage of the population with a positive MST living in an endemic area of leishmaniasis (Biagi 1953, Echandi 1953, Restrepo-Isaza 1980, Córdova-Uscanga et al. 1994, Arjona-Villicaña 2002). MST standardisation, safety, storage, longevity, sensitisation, sensitivity and specificity have been evaluated to support it as a reliable tool for epidemiological studies (Weigle et al. 1991, da Costa et al. 1996). Moreover, because the MST reflects the degree of cellular immunity and memory developed during *Leishmania* infection, the test has also been useful for evaluation of vaccination programs as an indicator of the protective immune response (Mayrink et al. 1979).

*Epidemiological studies of asymptomatic ACL infection* - Previous epidemiological studies conducted in endemic areas of ACL have reported findings of subjects with no history of ACL but with a positive MST. This MST-positive response has been attributed to prior asymptomatic infection with *Leishmania* spp. (Biagi 1953, Echandi 1953, Córdova-Uscanga et al. 1994, Arjona-Villicaña 2002).

González and Biagi (1968), in a study to document asymptomatic infection in humans, reported that five of six healthy volunteers inoculated with 500 promastigotes of *L. (L.) mexicana* developed an MST-positive response. The other volunteer developed a typical ulcerated skin lesion. The authors suggested that this type of infection could be playing an important role in the epidemiology of the disease and also considered that asymptomatic subjects may have developed protective immunity that could remain effective for years after the primary infection.

The most direct evidence supporting the existence of asymptomatic ACL infection comes from two prospective longitudinal studies (Weigle et al. 1993, Davies et al. 1995). In both studies, repeated MSTs were performed at intervals on cohorts of initially asymptomatic MST-negative persons. In the first study, done in Colombia, authors measured the incidence of asymptomatic infection by MST conversion as well as new cases of clinical infection by the development of new lesions. It was reported that 91% of primary infections were asymptomatic (Weigle et al. 1993). In the second study, conducted in the Peruvian Andes, the degree of acquired immunity in either asymptomatic or symptomatic infection was measured. As expected, symptomatic clinical infections led to protective immunity and relatively permanent MST responsiveness. However, the most important finding was that asymptomatic infection also led to protective immunity that positively correlated with MST induration size (Davies et al. 1995).

*Immunological studies of asymptomatic ACL infection* - It is generally accepted that the outcome of infection by *Leishmania* spp. depends mainly on the host immune response and parasite virulence. As previously noted, the outcomes of *Leishmania* infection range from asymptomatic to clinically overt disease. Asymptomatic ACL infection, as has been stated, is characterised by the absence of signs and symptoms. Accordingly, there is no evidence of tissue damage. This feature is suggestive that an immune response capable of controlling the parasite is elicited. Based on this argument, studies to characterise the immune response in ACL asymptomatic infection have been conducted.

Bosque et al. (2000) documented that in an endemic area of ACL in Colombia, the diameter of MST-positive induration was significantly higher among individuals with asymptomatic infection than those with clinical infection. Furthermore, linear regression analyses revealed a strong direct relationship between the production of IFN-g, TNF-α and IL-10 by peripheral blood mononuclear cells (PBMCs) among individuals with asymptomatic infection but not in those with clinical infection. The authors concluded that the identification of susceptible (clinically infected) and resistant (asymptomatically infected) individuals in circumstances of natural infection could provide a crucial basis for insightful investigation of the underlying mechanisms of susceptibility and resistance in human dermal leishmaniasis.

In an endemic area of ACL caused by *L. (V.) braziliensis* in Brazil, it was found that INF-g and TNF-a levels from lymphocyte supernatants in subjects with asymptomatic infection were significantly lower than they were in patients with active ACL (Follador et al. 2002). The authors suggested that IFN-g and TNF-a production in individuals with asymptomatic infection could be enough to control parasite growth and prevent tissue damage.

In Colombia, a comparative immunohistological analysis of the MST was performed to distinguish the cellular immune response to *Leishmania* between individuals with asymptomatic and those with clinical infection. CD8^+^ T lymphocytes and macrophages were found to be positively associated with MST biopsies from individuals with asymptomatic infection. The authors proposed that the MST provides a surrogate for the early response to *Leishmania* infection and that it therefore could be a useful tool to study and characterise immune protective mechanisms (Guarín et al. 2006).

Gomes-Silva et al. (2007) proposed that the absence of the disease in subjects with asymptomatic infection could be explained by their intrinsic ability to create a balance between immunoregulatory IL-10 and effector cytokine IFN-γ, leading to parasite control or destruction without producing skin tissue damage.

In a study to discern the interrelationship of T-cell differentiation and outcome of human infection, factors that regulate T-cell differentiation and Th1/Th2 cytokine responses in asymptomatic infection, active and historical chronic infection and recurrent cutaneous leishmaniasis were examined. By employing PBMCs, an inverse correlation was observed between GATA-3 expression and secretion of the proinflammatory cytokines INFg and TNFa in subjects with asymptomatic infection (Díaz et al. 2010).

T-cells with a regulatory phenotype and function (CD4+CD25+ cells) have been found in active ACL lesions (Campanelli et al. 2006, Bourreau et al. 2009). In a study to determine the role of these cell populations in the pathogenesis of chronic ACL by employing PBMCs, it was observed that CD4+CD25+ cells from individuals with asymptomatic infection have a higher capacity to suppress CD4 effector T cell IFN-g secretion than those from patients with chronic ACL (Rodríguez-Pinto et al. 2012).

The skin is the natural site of entry for infective promastigotes transmitted by sand flies obtaining a blood meal. Therefore, interaction with the dermal immune system at the initial stage of infection is likely to be important to disease outcome. Several studies have been conducted to characterise the *in situ* immune response against *Leishmania*. TNF-α, IL-lα, IL-10 and TGF-β were significantly increased in chronic compared to early lesions, suggesting a role of these cytokines in the immunopathogenesis of chronic disease (Melby et al. 1994). Moreover, both IL-10 and IL-12 mRNAs were co-expressed in most lesions of individuals with active LCL (Melby et al. 1996).

In a recent study, *in situ* gene expression of cytokines (IL-4, IL-10, IL-12, IFN-γ) and chemokines (MCP-1, MIP-1α) was examined in biopsies of *L. (L.) mexicana* active lesions. Additionally, MST biopsies were taken from subjects with healed lesions and those with asymptomatic infection. IL-12 and MCP-1 in the absence of IFN-γ gene expression were predominant in subjects with healed lesions and asymptomatic infections, suggesting that these molecules could play a role in early infection and skin-level outcomes (Valencia-Pacheco et al. 2014).

The frequency of CD8+ IFN-g+ cells after soluble *Leishmania* antigen stimulation was higher for asymptomatically infected individuals than for CL patients. The frequency of infected monocytes in cells from individuals with asymptomatic infection was lower than in cells from CL subjects (Cardoso et al. 2015).

## DISCUSSION

First, asymptomatic infection is the most common outcome after *Leishmania* spp. inoculation by infected *Lutzomya* in New World endemic areas of ACL. The epidemiological studies reviewed support the hypothesis that asymptomatic subjects with a positive MST after inoculation by *Leishmania-*infected sand flies are resistant and capable of controlling the infection without evidence of tissue damage. Nevertheless, the mechanisms involved in controlling the infection and responsible for the absence of clinical signs and symptoms remain to be identified. This knowledge is indeed relevant for better understanding the immune mechanisms that could be useful for vaccine development.

Epidemiological studies evaluating the incidence and prevalence of ACL have mostly focused on passive reporting systems. Commonly, only defined cases of ACL (*i.e.*, patients with active cutaneous lesions) are recorded. Therefore, it is important to note that ACL infection includes not only active lesions but also asymptomatic infection, reinfection and reactivation. Accordingly, there is an underestimation of the prevalence of ACL. The number of detected cases does not represent the number of newly acquired *Leishmania* infections. The rates of newly acquired asymptomatic and clinical infections are essential for estimating the potential impact of transmission control measures. Moreover, it is necessary to evaluate the role of asymptomatic infected individuals as potential parasite reservoirs and to evaluate the risk of transmission through blood donation or vertical transmission.

A positive MST is the most important feature of ACL asymptomatic infection. Epidemiological studies highlighted that MST induration size is significantly higher in asymptomatic infection when compared with clinical infection (Bosque et al. 2000, Follador et al. 2002, Trujillo et al. 2002). Moreover, induration size positively correlates with immune protection and the presence of CD8+ T lymphocytes and macrophages (Davies et al. 1995, Cardoso et al. 2015). Therefore, MST could be a good biomarker of resistance and a useful tool for assessing the immune response in asymptomatic individuals. Regardless of the great advances in molecular technology, to date, there is no knowledge related to the antigens responsible for inducing the MST response in leishmaniasis. This knowledge could be of great importance for vaccine strategies.

It is accepted worldwide that the outcome of infection depends on the host immune response and *Leishmania* species. Most of our knowledge regarding the protective and pathogenic immune response against *Leishmania* has been obtained by studying active clinical infection (acute or chronic). Nonetheless, we know that the spectrum of disease ranges from asymptomatic infection to manifest clinical disease. However, the question to be answered remains: *Which mechanisms are responsible for inducing ACL asymptomatic infection?*

It is well known that a crucial aspect of defence against *Leishmania* is the ability of the host to mount a cell-mediated immune response capable of controlling the parasite. Knowledge about the composition of the cell populations recruited in the early phase of the infection is essential for defining infection outcomes. The signals that initiate and regulate the early immune response and local accumulation of cell subsets in the skin are not known and understood well in asymptomatic infection. This knowledge is critical for the development of vaccine candidates and possibly to achieve control of ACL in the near future.

Cytokines play a critical role in shaping the host immune response to *Leishmania* infection and directing the development of protective and non-protective immunities during infection (Cummings et al. 2010). The paradigm for the Th1/Th2 dichotomy is largely based on the curative/non-curative responses to *L. major* infection in the murine model. Resolution of murine *L. major* infection is associated with the capacity of Th1 cells to generate IFN-g and TNF-a. Exacerbation and progression of disease are associated with the activation of Th2 cells and production of IL-4, IL-5 and IL-10. The Th1/Th2 dichotomy observed in murine cutaneous leishmaniasis has not been demonstrated in humans. Therefore, factors governing the host immune response and disease outcome during leishmaniasis must be more complex in human than in mice (Tacchini-Cottier et al. 2012).

IFN-γ secreted by CD4+ Th1 cells synergises with TNF-a to activate macrophage leishmanicidal activity (Gollob et al. 2014). Thus, IFN-γ plays a critical role in the control of the infection. This previously reported knowledge based on the *L. major* mouse model has gained keen insights. However, IFN-γ has now been identified as a mediator of tissue damage in chronic ACL (Soong et al. 2012). Therefore, it also participates actively in the immunopathogenesis of chronic lesions as a proinflammatory cytokine.

The role of TNF-α in host resistance to infection by *L. major* has been known since the 1990s when its protective effect was suspected to relate to its ability to activate macrophage leishmanicidal activity (Liew et al. 1990). Since then, the role of TNF-a has been established as providing protective immunity against *L. major* in the experimental murine model (Wilhelm et al. 2005). Moreover, TNF-α is known to play a key role in granuloma formation (Roach et al. 2002). Through studies employing anti-TNF-a drugs against rheumatoid arthritis, it has been documented that these drugs can favour the development or reactivation of infections by obligate intracellular microorganisms (Feldmann & Maini 2001). TNF-a is a key cytokine involved in Th1-dependent granuloma formation, thus explaining why patients treated with TNF antagonists have an increased risk of granulomatous infectious disease (Zanger et al. 2012).

Il-10 was identified in the *L. major* mouse model as a potent inhibitor of cytokines produced by both Th1 lymphocytes and monocytes that favours the development of a Th2 type immune response that leads to exacerbation and progression of the disease. However, this knowledge could not be extrapolated to other *Leishmania* spp. and humans. Updated evidence supports an important role of IL-10 as a regulatory cytokine (Gollob et al. 2014).

Early events in host-parasite interactions are likely to influence the future course of the disease. After dermal infection with *Leishmania*, a local inflammatory process is initiated involving the accumulation of leukocytes at the site of parasite delivery. The composition of the cell populations recruited in this early phase of infection seems to be essential for defining the outcome of the disease. Members of the chemokine family have fundamental roles in attracting specific subsets of leukocytes to the site of infection and then stimulating them (Teixeira et al. 2006).

The chemokine system has a well-known role in orchestrating immune cell migration and positioning within an organism (Griffith et al. 2014). MCP-1 has been noted as an important chemokine in the early events of *Leishmania* infection (Ritter et al. 1996). MCP-1 in the absence of IFN-γ gene expression was predominant in subjects with healed lesions and asymptomatic infection, suggesting that this molecule could play a role in early infection and skin-level outcomes (Valencia-Pacheco et al. 2014).

CD4+ CD25+ T cells, named regulatory T cells (Tregs), are responsible for maintaining self-tolerance. In infectious diseases, Tregs also regulate the intensity and duration of immune responses, limiting damage to host tissues. The CD4+CD25+ T cells that accumulated in the skin lesions of the patients infected with *L. (V.) braziliensis* exhibited phenotypic and functional characteristics of natural Tregs, suggesting that they contribute to the local control of effector T cell functions (Campanelli et al. 2006).

The role of CD8^+^ T cells in human CL is not fully explained. A protective or deleterious role of CD8^+^ T cells in human cutaneous leishmaniasis (CL) has been debated. These cells are generally considered to contribute to immunity and protection against *Leishmania* (Müller et al. 1991). However, the role of CD8+ granzyme B^+^ T-cells mediating tissue injury in CL by *L. braziliensis* has been documented (Santos et al. 2013).

Altogether, the immunological studies reviewed have enhanced our knowledge about some of the regulatory mechanisms involved in controlling parasite and tissue damage in asymptomatic ACL infection. It is not surprising that regulatory molecules (Il-10, TNF-a) and cells (CD4+CD25+, CD8+ IFN-g+) have been identified as possible determinant factors. Undoubtedly, identification of immunological markers responsible for the development of asymptomatic infection is a great challenge. Taking into account the great complexity of host cells and molecules involved, it is certain that more efforts will be required to clarify this issue.

In conclusion, there is evidence that (i) asymptomatic infection is the most common outcome in endemic areas of ACL; (ii) MST is a good biomarker of resistance in subjects with ACL asymptomatic infection; (iii) MST provides a surrogate for early response to *Leishmania* infection, and therefore, it could be a useful tool to study and characterise the immune response elicited in ACL asymptomatic infection; and (iv) immunological studies support the hypothesis that there is an immune response capable of controlling the parasite and preventing tissue damage.

Although data supporting the existence of asymptomatic ACL infection existence were obtained through MST standardisation, there is limited knowledge regarding its epidemiological and immunological significance. Several important questions remain to be answered: (A) What is the prevalence and incidence of asymptomatic ACL infection in endemic areas? (B) What is its role in transmission? (C) Are individuals with asymptomatic ACL infection potential reservoirs of the parasite? (D) Should a survey be conducted of the population at risk of developing the disease because of immunosuppression? (E) Is there a risk of transmission through blood donation or vertical transmission? (F) What are the key factors responsible for induction of asymptomatic ACL infection? (G) What are the biomarkers of the immune response elicited in asymptomatic ACL infection? (H) Could identification of these biomarkers could be useful for vaccine development?
